# Liver Stiffness Decreases Rapidly in Response to Successful Hepatitis C Treatment and Then Plateaus

**DOI:** 10.1371/journal.pone.0159413

**Published:** 2016-07-21

**Authors:** Sweta Chekuri, Jillian Nickerson, Kian Bichoupan, Roberta Sefcik, Kamini Doobay, Sanders Chang, David DelBello, Alyson Harty, Douglas T. Dieterich, Ponni V. Perumalswami, Andrea D. Branch

**Affiliations:** 1 Division of Liver Diseases, Icahn School of Medicine at Mount Sinai, New York, NY, United States of America; 2 Department of Medical Education, Icahn School of Medicine at the Mount Sinai Medical Center, New York, NY, United States of America; Saint Louis University, UNITED STATES

## Abstract

**Background and Aim:**

To investigate the impact of a sustained virological response (SVR) to hepatitis C virus (HCV) treatment on liver stiffness (LS).

**Methods:**

LS, measured by transient elastography (FibroScan), demographic and laboratory data of patients treated with interferon (IFN)-containing or IFN-free regimens who had an SVR24 (undetectable HCV viral load 24 weeks after the end of treatment) were analyzed using two-tailed paired t-tests, Mann-Whitney Wilcoxon Signed-rank tests and linear regression. Two time intervals were investigated: pre-treatment to SVR24 and SVR24 to the end of follow-up. LS scores ≥ 12.5 kPa indicated LS-defined cirrhosis. A p-value below 0.05 was considered statistically significant.

**Results:**

The median age of the patients (n = 100) was 60 years [IQR (interquartile range) 54–64); 72% were male; 60% were Caucasian; and 42% had cirrhosis pre-treatment according to the FibroScan measurement. The median LS score dropped from 10.40 kPa (IQR: 7.25–18.60) pre-treatment to 7.60 kPa (IQR: 5.60–12.38) at SVR24, p <0.01. Among the 42 patients with LS-defined cirrhosis pre-treatment, 25 (60%) of patients still had LS scores ≥ 12.5 kPa at SVR24, indicating the persistence of cirrhosis. The median change in LS was similar in patients receiving IFN-containing and IFN-free regimens: -1.95 kPa (IQR: -5.75 –-0.38) versus -2.40 kPa (IQR: -7.70 –-0.23), p = 0.74. Among 56 patients with a post-SVR24 LS measurement, the LS score changed by an additional -0.90 kPa (IQR: -2.98–0.5) during a median follow-up time of 1.17 (IQR: 0.88–1.63) years, which was not a statistically significant decrease (p = 0.99).

**Conclusions:**

LS decreased from pre-treatment to SVR24, but did not decrease significantly during additional follow-up. Earlier treatment may be needed to reduce the burden of liver disease.

## Introduction

Hepatitis C Virus (HCV) is a leading cause of liver cirrhosis, end stage liver disease, and hepatocellular carcinoma (HCC) [1–3]. By 2007, the age-adjusted mortality for HCV was higher than for human immunodeficiency virus (HIV) in the United States [4]. The goals of HCV treatment are to achieve a sustained virological response (SVR), to halt the progression of liver damage, and to establish conditions that may allow hepatic fibrosis to regress. Hepatic fibrosis stage is a predictor or liver disease progression during chronic infection. Successful treatment reduces fibrosis and liver-related mortality [5–9]. Most studies show that HCV cure diminishes HCC risk [10–13], but a recent study had contrasting findings [14, 15]. While fibrosis regresses in most patients post-SVR, repair is frequently incomplete and cirrhosis persists in about 40% of patients who had cirrhosis prior to treatment [16–20].

To evaluate the impact of HCV treatment on liver damage, serial measurements of liver fibrosis must be obtained. Transient elastography (FibroScan, Echosens, Paris, France) is an attractive method for obtaining repeated measurements because it eliminates the pain, morbidity, and mortality that can accompany liver biopsy [21]. In a meta-analysis, Talwalkar et al. found that transient elastography has a 91% specificity and an 87% sensitivity for detecting cirrhosis compared to liver biopsy [22]. Over the years, several studies have reported a decrease in post-treatment liver stiffness (LS) scores compared to pre-treatment [8, 9, 16, 23–25]. A recent study of 93 patients who achieved an SVR conducted by Arima et al. reported a decrease in median LS scores from 8.0 kilopascals (kPa) [Interquartile range (IQR): 5.0–11.9] pre-treatment to 5.3 kPa (IQR: 4.1–6.3) two years after the end of treatment (EOT), (P< 0.01) [9]. Hezode and colleagues had similar findings and reported a median change in EOT, SVR12 and SVR24 LS score compared to pre-treatment as -2.0 (-3.6–0.5), -2.4 (-2.4–1.0) and -3.4 kPa (-4.7 –-1.1), respectively. Hezode et al. attributed the decrease in LS that occurred during the time interval between baseline and SVR to a reduction in liver inflammation [23]. Karlas et al. was the only group to investigate IFN-free treatment regimens. In patients with advanced fibrosis or cirrhosis they observed a median decrease in LS from 32 kPa at pre-treatment to 20 kPa at SVR12 [25]. To our knowledge, our study is the first to compare LS changes in patients treated with interferon (IFN)-containing and IFN-free regimens.

## Material and Methods

### Study Design and Population

This observational study analyzed data on 100 patients with chronic HCV infection who achieved an SVR24 and who underwent FibroScan before and after HCV treatment during the time period of 2008 to 2016 at the Mount Sinai Medical Center in New York, NY. Patients with HCV were identified by querying the Mount Sinai data warehouse for patients whose record included the International Classification of Disease, Ninth Revision (ICD-9) code for chronic HCV. The Icahn School of Medicine at Mount Sinai Institutional Review Board approved this study, GCO# 10–0032. The inclusion criteria included the presence of chronic HCV infection with a detectable HCV viral load in the blood prior to treatment; documented evidence of SVR24, i.e., undetectable HCV viral load in the blood a minimum of 24 weeks after the end of treatment [26]; and FibroScan measurements obtained prior to treatment and after achieving SVR24. Patients were excluded if they did not achieve an SVR24, if they had HIV co-infection, or had undergone liver transplantation.

Medical record data were obtained on FibroScan measurements, body mass index (BMI), hemoglobin (Hb), platelet count, international normalized ratio (INR), creatinine, alanine aminotransferase (ALT), aspartate aminotransferase (AST), alkaline phosphatase (ALP), ɣ-glutamyl-transpeptidase (GGT), total bilirubin, and alpha fetoprotein (AFP) prior to treatment and after achieving SVR24. Some records lacked data about albumin, INR, GGT or AFP at one or more time points, as indicated in the tables. The FIB-4 index was used to estimate the stage of liver fibrosis. It was calculated as: Age (years) × AST (U/L)/(Platelet count (10^9^/L) × (ALT^1/2^ (U/L) [27]. A FIB-4 score value ≥ 3.25 signified advanced fibrosis/cirrhosis. Antiviral regimens used to treat patients were categorized as IFN-containing and IFN-free. IFN-containing regimens included IFN/ribavirin (RBV) with or without telaprevir (TVR) or boceprevir (BOC). IFN-free regimens included sofosbuvir (SOF)/ribavirin (RBV), SOF/simeprevir (SMV), SOF/RBV/SMV, and SOF/ledipasvir (LDV).

### Liver Stiffness Measurement

FibroScan was performed on the right lobe of the liver by trained clinicians at Mount Sinai Hospital. A total of 10 measurements, expressed in kPa, were obtained at each assessment and the median was determined [28]. LS score range from 2.50 to 75 kPa [28, 29]. LS values were used to estimate the METAVIR fibrosis stage as follows: F0-F1: 2.5 to 6.9 kPa; F2: 7.0 to 9.4 kPa; F3: 9.5 to 12.4 kPa; F4: ≥ 12.5 kPa. Cirrhosis was defined as an LS score of 12.5kPa or more [30]. Data were analyzed for two time intervals: pre-treatment to the first FibroScan result obtained ≥ 24 weeks after the EOT, which was used as the SVR24 score, and SVR24 to the end of follow-up.

### Statistical Methods

Continuous variables were expressed as the median and IQR. Categorical variables are depicted as absolute numbers and percentages. Data were analyzed using two-tailed paired t-tests, Mann-Whitney Wilcoxon signed-rank tests, Fisher’s exact test and linear regression. A p-value below 0.05 was considered statistically significant.

## Results

### Pre-Treatment Characteristics

LS was analyzed in 100 patients (Table 1). The pre-treatment LS measurements ranged from 4.4 to 72.0 kPa with a median of 10.40 kPa (IQR: 7.25–18.60). Based on the LS scores, the estimated METAVIR fibrosis stage distribution was 23% F0-F1, 21% F2, 14% F3 and 42% F4 (cirrhosis). Prior to treatment, patients had a median age of 60 years (IQR: 54–64). Seventy-two percent were male and 60% were Caucasian. The distribution of HCV genotypes was 85% genotype 1, 10% genotype 2, and 5% genotype 3.

**Table 1:** Pre-treatment Patient Characteristics.

Characteristic	Total Patients
Number	N = 100
Gender, Male	72
Age (Years)	60 (54–64)^a^
BMI kg/m^2^	25.8 (23.3–28.6)
Race	
Caucasian	60
African–American	9
Other	31
History of Smoking (N = 99)	64
History of Alcohol use (N = 96)	70
Laboratory Tests	
Glucose (65–139 mg/dL) ^b^	90 (83.5–106.5)
Hemoglobin–g/dL (13.9–16.3 g/dL)	14.5 (13.5–15.6)
Platelets–x 10^3^ (150–450 cells/μL)	157 (130–195)
INR (N = 73)	1 (1–1.1)
Creatinine (0.6–1.4 mg/dL)	0.96 (0.82–1.1)
Total bilirubin (0.1–1.2 mg/dL)	0.7 (0.5–0.9)
AST (1–50 U/L)	58 (38–94)
ALT (1–53 U/L)	72 (46–125)
AST/ALT	0.8 (0.7–1.0)
ALP (44–147 IU/L)	77 (60–101)
GGT (N = 99) (0–51 IU/L)	62 (32–96)
AFP (N = 86) (0–9.0 ng/mL)	5.3 (3.0–9.3)
Albumin (3.5–4.9 g/dL)	4.4 (4.1–4.5)
Hemoglobin A1c (N = 84)	5.4 (5.2–5.9)
Genotype	
Genotype 1	85
Genotype 2	10
Genotype 3	5
Treatment Regimens	
IFN–containing regimens	52
IFN–free regimen	48
Estimated METAVIR Stage Stratified by FibroScan	
F0-F1	23
F2	21
F3	14
F4	42
FIB– 4 Score	
0–1.25	12
1.25–3.25	56
>3.25	32
FibroScan score (kPa)	10.40 (7.25–18.60)

^a^All values reported as median (interquartile range 1–3).

^b^Normal ranges indicated in parentheses.

BMI, body mass index; INR, international normalized ratio; AFP, alpha fetoprotein; AST, aspartate aminotransferase; ALT, alanine aminotransferase; ALP, alkaline phosphatase; GGT, ɣ-glutamyl-transpeptidase; IFN, interferon; SMV, simeprevir; SOF, sofosbuvir; RBV, ribavirin; LDV, ledipasvir.

### The Impact of SVR24 on Liver Stiffness and Estimated Fibrosis Stage

**Fig 1:**
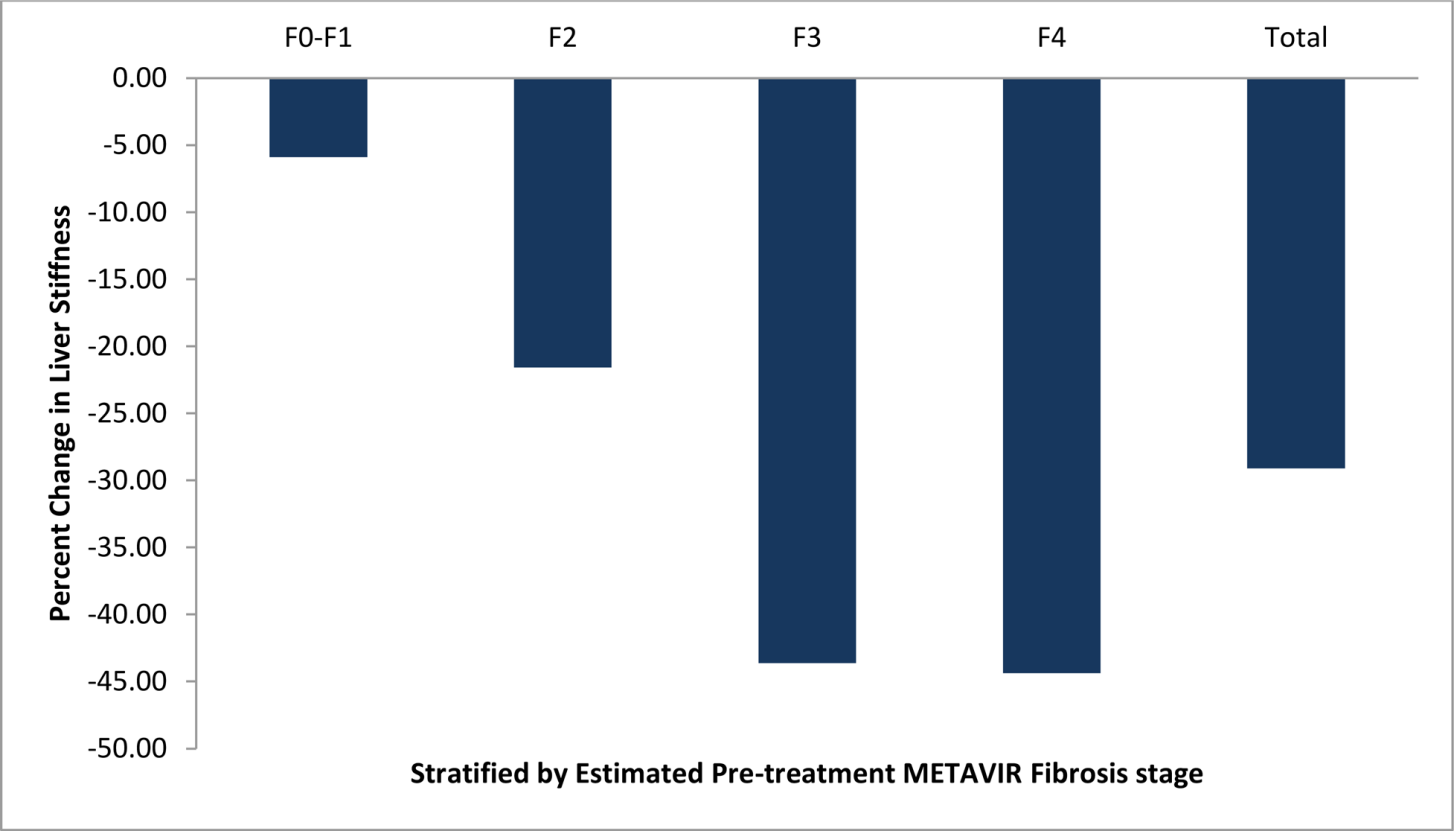
Percent changes of pre-treatment versus SVR24 liver stiffness liver. The figure shows the median percent change of LS for each METAVIR fibrosis stage stratified by the pre-treatment LS score.

**Fig 2:**
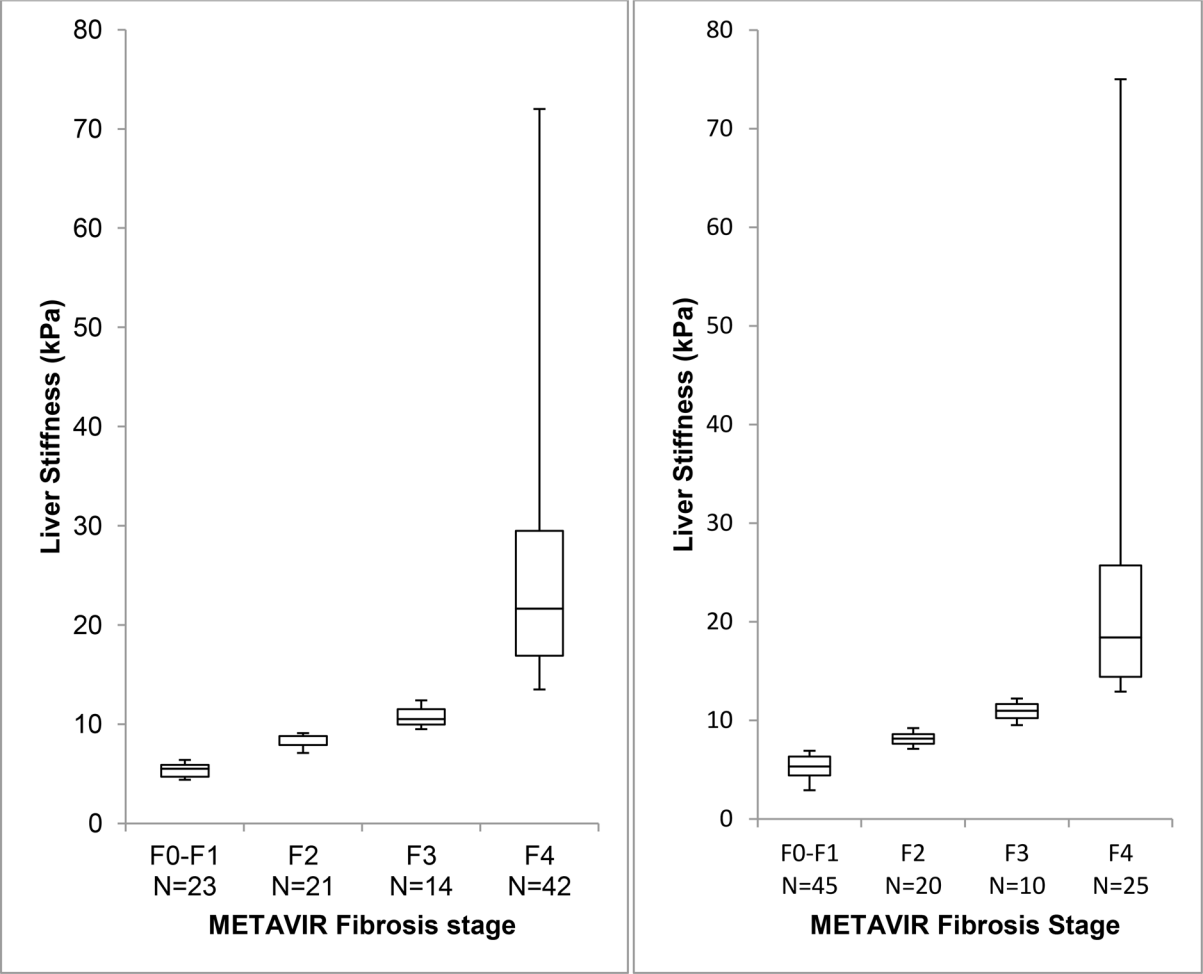
Pre-treatment versus SVR24 liver stiffness. Comparison of pre-treatment to SVR24 LS in kPa stratified by METAVIR fibrosis stage with (A) depicting median and interquartile ranges for pre-treatment scores and (B) depicting median and interquartile ranges for SVR24 scores. The vertical axis is the liver stiffness in kPa and the horizontal axis is the METAVIR fibrosis stage and the number of patients in each fibrosis stage. The top and bottom boxes represent interquartile ranges (interquartile 1 and interquartile 3 respectively). The lines through the boxes represent median values. The whiskers at the end of the box represent the maximum and minimum values.

The median pre-treatment and SVR24 LS scores were 10.40 kPa (IQR: 7.25–18.60) and 7.60 kPa (IQR: 5.60–12.36), a statistically significant change of -2.15 kPa (IQR: -6.68 –-0.3), p<0.01. The median percent change was -29% (IQR: -48 –-3) (Fig 1). The median follow-up time from pre-treatment to the SVR24 FibroScan was 1.71 years (IQR: 1.28–2.57). Changes in estimated METAVIR fibrosis stage stratified by the pre-treatment stiffness score are presented in Tables 2 and 3, and Fig 2. The median change was similar in males and females and did not differ between the two: -2.30 kPa (IQR: -6.68 –-0.08) versus -2.10 kPa (IQR: -6.53 –-0.93), p = 0.5, for the comparison of the change in males and females. The median change in patients with cirrhosis was almost 5-fold greater than in patients without cirrhosis: -7.10 kPa (IQR: -12.10 –-0.98 kPa) versus -1.65 kPa (IQR: -3.0 –-0.23 kPa), p<0.01. The median percent change was also greater for patients who had cirrhosis at pre-treatment: -44% (IQR: -53 –-4.4) versus -20% (IQR: -35 –- 3) in patients without pre-treatment cirrhosis, p<0.01. Despite the favorable changes that occurred in many patients, 25 of 42 patients (60%) who were cirrhotic pre-treatment remained cirrhotic at SVR24. Only 26 (34%) out of 77 patients with a fibrosis stage >F2 (estimated fibrosis stage using FibroScan) decreased to stage F0-F1 at SVR24.

**Table 2:** Estimated Fibrosis Stage using Fibroscan at SVR24 Stratified by the Pre-treatment Stiffness Score.

Pre-treatment	SVR24 Score	SVR24 Score	SVR24 Score	SVR24 Score
Median (IQR)	F0—F1 <7kPa	F2 >7–9.4 kPa	F3 9.5–12.4 kPa	≥ 12.5 kPa
**Total Group**				
**N = 100**	-	-	-	-
10.4 (7.3–18.6)				
**F0—F1 (N = 23)**	19	3	1	0
5.5 (4.7–5.9)^a^	4.8 (4.2–5.9)	7.6 (7.5–8.4)	(11.2)	
**F2 (N = 21)**	13	6	2	0
8.8 (7.9–8.8)	5.9 (4.8–6.4)	8.3 (7.7–8.6)	9.9 (9.7–10.1)	
**F3 (N = 14)**	11	3	0	0
10.5 (10.0–11.5)	6.1 (4.7–6.3)	8.6 (8.6–8.7)		
**F4 (N = 42)**	2	8	7	25
21.7 (16.9–29.5)	6.3 (5.9–6.6)	7.8 (7.6–8)	10.1 (10.0–10.1)	14.7 (14.6–14.9)

^a^All values reported as median (interquartile range 1–3).

**Table 3:** Median Change in LS from Pre-treatment to SVR24 Stratified by Pre-treatment Estimated Fibrosis Stage using FibroScan.

Pre-treatment Metavir Stage	Median Pre-Treatment Score	Median SVR24 score	Median Change	p Value
**F0—F1 (N = 23)**	5.5 (4.7–5.9)^a^	4.9 (4.3–6.8)	-0.3 (-1.35–1.35)	0.6
**F2 (N = 21)**	8.8 (7.9–8.8)	6.7 (5.4–8.1)	-1.7 (-2.6 –-0.4)	<0.01
**F3 (N = 14)**	10.5 (10.0–11.5)	6.3 (5.0–6.9)	-4.6 (-5.6 –-3.3)	<0.01
**F4 (N = 42)**	21.7 (16.9–29.5)	14.0 (10.0–21.2)	-7.1 (-12.1 –-1.0)	<0.01
**Total N = 100**	10.4 (7.3–18.6)	7.6 (5.6–12.4)	-2.2 (-6.7 –-0.3)	<0.01

^a^All values reported as median (interquartile range 1–3).

Patients were treated with IFN-containing regimens (52%) or with IFN-free regimens (48%). The median follow-up from pre-treatment to SVR24 in patients treated with IFN-containing and IFN-free regimens was 2.3 years (IQR: 1.8–3.2) and 1.3 years (1.0–1.6), respectively. The median change in patients treated with IFN-containing regimens was -1.95 kPa (IQR:-5.75 –-0.38 kPa), p<0.01, and the median change in patients treated with IFN-free regimens was -2.40 kPa (IQR:-7.70 –-0.23 kPa), p<0.01. The difference in median change in LS between the IFN-containing regimens and IFN-free regimens was not statistically significant, p = 0.74. (Table 4)

**Table 4:** Changes in Liver Stiffness (pre-treatment to SVR24): IFN vs IFN-free Regimens.

	IFN-regimens (N = 52)	IFN-free regimens (N = 48)	p value
**Median time from Pre-treatment to SVR24 (years)**	2.3 (1.8–3.2)^a^	1.3 (1.0–1.6)	-
**Change in LS (kPa)**	-2.0 (-5.8 –-0.4)	-2.4 (-7.7 –-0.2)	0.7
**Percent change in LS score**	-30 (-48 –-4)	-27 (-47 –-2)	0.8

^a^All values reported as median (interquartile range 1–3).

### The Impact of SVR on BMI and Clinical Laboratory Values

Differences in pre-treatment and SVR24 values were calculated for BMI, Hb, platelets, creatinine, albumin, INR, AFP, total bilirubin, AST, ALT, ALP, and GGT (Table 5). BMI increased slightly in patients treated with both IFN-containing regimens (N = 52) and IFN-free regimens (N = 48): median change of 0.47 kg/m^2^ (IQR: -0.33–2.04), p = 0.02 and 0.69 kg/m^2^ (IQR: -0.18–1.58), p<0.01, respectively. For the total group, BMI (N = 98) increased from 25.82 kg/m^2^ (IQR 23.35–28.63) to 26.68 kg/m^2^ (IQR 24.41–29.16), a statistically significant median change of 0.67 kg/m^2^ (IQR: -0.32–1.89), p<0.01.

**Table 5:** Comparison of Pre-treatment and SVR24 Clinical Laboratory Values.

Variables (N = 100)	Pre-treatment	SVR24	Change	p-Value^a^
Hemoglobin–g/dL (13.9–16.3 g/dL)^b^	14.5 (13.5–15.6)^c^	14.6 (13.4–15.4)	-0.1 (-0.7–0.5)	0.3
Platelets–x 10^3^ (150–450 cells/μL)	157 (130–195)	180 (140–206)	13 (-7–36)	<0.01
Platelets <150 x 10^3^ cells/μL	44%^d^	27% ^d^	-17% ^d^	<0.01
Creatinine (0.6–1.4 mg/dL)	1 (0.8–1.1)	1 (0.8–1.1)	0 (-0.1–0.1)	0.08
Albumin (N = 98) (3.5–4.9 g/dL)	4.4 (4.1–4.5)	4.5 (4.2–4.6)	0.1 (-0.2–0.3)	<0.03
Albumin <3.5g/dL	4%^d^	0% ^d^	-4% ^d^	0.01^e^
INR (N = 42)	1 (1–1.1)	1 (0.9–1.0)	0 (-0.1–0)	<0.02
AFP (N = 86) (0–9.0 ng/mL)	5.3 (3–9.3)	3 (2.1–4.3)	-2 (-4.7 - -0.6)	<0.01
Total bilirubin (0.1–1.2 mg/dL)	0.7 (0.5–0.9)	0.6 (0.4–0.8)	-0.1 (-0.2–0.01)	0.2
Bilirubin >1.2 mg/dL	11%^d^	5% ^d^	-6%^d^	0.3^e^
AST (1–50 U/L)	58 (38–94)	23 (20–31)	-34 (-65–14)	<0.01
ALT (1–53 U/L)	72 (46–125)	20 (17–27)	-52 (-101–23)	<0.01
AST/ALT	0.8 (0.7–1.0)	1.2 (0.9–1.4)	0.3 (0.1–0.6)	<0.01
ALP (44–147 IU/L)	77 (60–101)	70 (55–90)	-6 (-17–3)	<0.01
GGT (N = 92) (0–51 IU/L)	62 (32–96)	24 (17–41.5)	-28 (-61–5)	<0.01
FIB-4 score	2.3 (1.6–3.8)	1.8 (1.2–2.6)	-0.6 (-1.5–0.18)	<0.01

^a^Mann-Whitney test unless indicated

^b^Normal ranges indicated in parentheses following lab test name

^c^Values reported as median (interquartile range 1–3) unless stated

^d^Values reported as percent of patients

^e^Fisher’s exact test

INR, international normalized ratio; AFP, alpha fetoprotein; AST, aspartate aminotrasferase; ALT, alanine aminotransferase; ALP, alkaline phosphatase; GGT, ɣ-glutamyl-transpeptidase.

SVR was accompanied by statistically significant increases in platelets, p<0.01, and albumin, p<0.03, and decreases in AST, ALT, ALP and GGT, p<0.01. Hb decreased, but the change was not significant, p = 0.27. Seventeen of 44 patients with pre-treatment platelet counts below the lower limit of normal (150 x 10^3^ cells/μL) had an SVR24 platelet count in the normal range, significantly decreasing the percentage of the population with thrombocytopenia, p<0.01. The FIB-4 score decreased from 2.34 (IQR: 1.61–3.76) to 1.80 (IQR: 1.22–2.57), a statistically significant median change of -0.57 (IQR: -1.55–0.18), p<0.01. There was a significant association between the decrease in AST and ALT and the percentage decrease in LS, p<0.01 (Table 6). Among 86 patients with data available, AFP decreased from 5.30 ng/mL (IQR: 3.0–9.33) to 3.0 ng/mL (IQR: 2.12–4.27), a statistically significant change of -2.0 ng/ml (IQR: -4.70 –-0.63), p <0.01.

**Table 6:** Linear Regression Analysis of the Percent Change in Liver Stiffness and the Change in Laboratory Values from Pre-treatment to SVR24.

Variable (N = 100)	p-value	R^2^
Hemoglobin	0.5	0.002
Platelets	0.7	0.075
Bilirubin	0.7	0.005
Albumin (N = 98)	0.5	0.021
ALT	<0.01	0.049
AST	<0.01	0.070
AFP (N = 86)	0.3	0.237

AST, aspartate aminotrasferase; ALT, alanine aminotransferase; AFP, alpha fetoprotein.

### Changes in Liver Stiffness during Post-SVR Follow-Up

**Fig 3:**
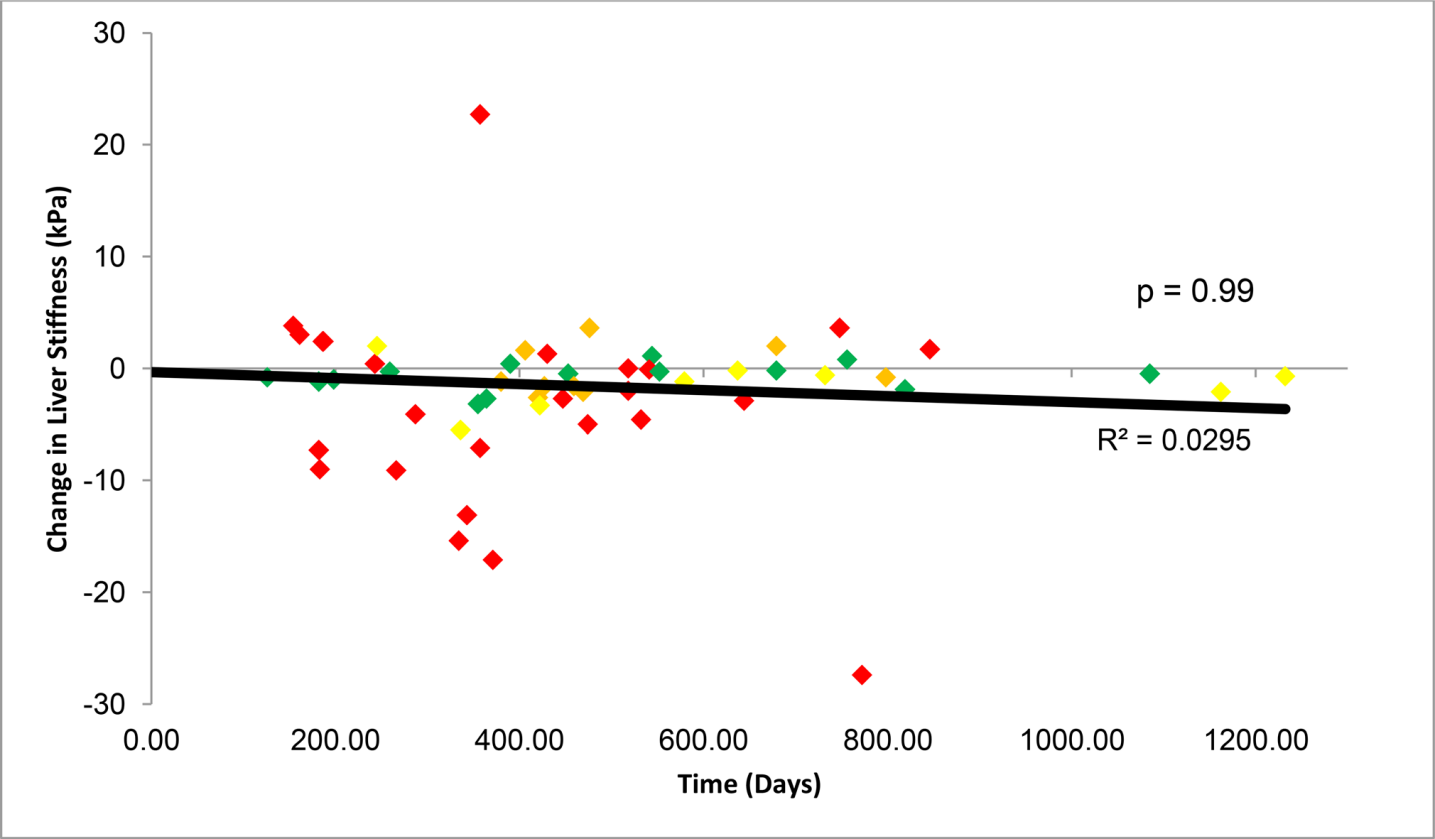
Liver stiffness changes during follow-up post-SVR24. The graph shows FibroScan scores in 56 patients who had an additional measurement afterSVR24. 0 on the horizontal axis indicates SVR24. Stiffness decreased over time, but the change was not significant as demonstrated by linear regression with a p-value = 0.9. The colors indicate the pre-treatment fibrosis stage as estimated from the LS score, green (F0-1), yellow (F2), orange (F3), red (F4).

Fifty-six of the 100 patients had LS measurements in the follow-up period after SVR24. The median follow-up time after the SVR24 measurement was 1.17 years (IQR: 0.88–1.63). Among these 56 patients, the median pretreatment, SVR 24, and the last follow-up LS scores were 12.70 kPa (8.30–21.15), 8.35 kPa (IQR: 6.68–14.03) and 7.70 kPa (IQR: 5.30–11.60), respectively. The median LS change from SVR24 to the last follow-up was -0.9 kPa (IQR: -2.98–0.5), which was not statistically significant, p = 0.9 (Fig 3). Among these 56 patients, 28 had LS scores in the cirrhotic range prior to treatment. Sixteen of the 28 patients continued to have LS scores in the cirrhotic range at SVR24, but five had scores that fell into the non-cirrhotic range during follow-up. Conversely, one patient with an LS score in the non-cirrhotic range at SVR24 had a score in the cirrhotic range during post-SVR24 follow-up. Thus, during the post-SVR24 follow-up period, the percentage of patients with cirrhosis changed from 16/56 (29%) to 12/56 (21%), an 8% decrease in the percentage of the population with cirrhosis that was not statistically significant, p = 0.99.

## Discussion

This observational study of 100 patients was performed to investigate the impact of successful HCV treatment on liver stiffness, a composite indicator of liver fibrosis, inflammation and edema [31]. HCV virological cure was accompanied by a significant decrease in LS, consistent with the findings of previous studies [8, 9, 23, 24]. To our knowledge, our study is the first to compare LS changes in patients treated with IFN-containing vs. IFN-free regimens, in men vs. women, and in cirrhotics vs. non-cirrhotics. We found no significant difference in the decrease in LS in patients treated with IFN-containing versus IFN-free regimens and no significant difference between men and women. In contrast, the median change in LS of patients with cirrhosis was nearly 5-fold greater than that in non-cirrhotics and the median percent change was significantly higher in the cirrhotics: -44% versus -20%, p<0.01. Despite the improvement, 60% of patients with cirrhosis at pre-treatment continued to have cirrhosis at SVR24. This result is consistent with previous studies, which established that cirrhosis often persists in patients who achieve an SVR [16–20]. The failure of SVR to resolve cirrhosis in a large percentage of patients indicated that patients must be identified and transitioned into treatment before permanent liver damage has occurred. Otherwise, the maximum benefit of anti-HCV treatment will be missed.

For the group as a whole, the magnitude of the median change in LS (pre-treatment to SVR24) from 10.40 kPa to 7.60 kPa (p<0.01), was consistent with the results of Martinez et al., which showed a significant decrease in mean LS from 10.6 kPa to 8.5 kPa (p<0.01) [32] and those of Arima etal, which showed a median decrease (pre-treatment to SVR48) from 8.0 kPa (IQR: 5.0–11.9) to 5.3 kPa (IQR: 4.2–7.0), p<0.01 [9]. Arima et al. followed patients for an additional two years after EOT and found that the median LS score was stable: 5.3 kPa (IQR: 4.2–7.0) versus 5.3 kPa (IQR: 4.1–6.3). Consistent with these findings, our study revealed that LS did not change significantly over a greater than one year follow-up period post-SVR24.

The improvement in AST, ALT, platelets and albumin levels with SVR is well established, but LS score changes in comparison to these parameters have not been thoroughly examined. Our study showed significant improvement in platelet counts (pre-treatment to SVR24). Thirty-nine percent of patients with pre-treatment platelet counts below the lower limit of normal had values in the normal range at SVR24, which was consistent with previous data [33–35]. AST, ALT, GGT and AFP decreased in our study, and this is also consistent with prior reports [9, 33–37]. Compared to pre-treatment, we found a significant increase in albumin levels (p<0.03) at SVR24 and all four patients with pre-treatment albumin levels below 3.5mg/dL had values in the normal range at SVR24. Deterding et al. also reported improved albumin levels at SVR12 (p<0.01) and described a marked increase in albumin levels at SVR12 in patients with a pre-treatment albumin below 3.5g/dL, consistent with our findings [33]. In our study, we found a non-significant decrease in bilirubin levels (p<0.2), differing somewhat from Deterding et al. who reported a significant decrease in bilirubin at SVR12 (p<0.01) [33]. This difference could be attributed to the lower pre-treatment bilirubin in our study compared to Deterding et al. Finally, we found that the decreases in AST and ALT levels correlated with the percent change of LS, p<0.01.

A recent study showed a significant decrease in hemoglobin (p = 0.02) [33], while we found a non-significant median decrease in hemoglobin levels (p = 0.3). This discrepancy might have occurred because 100% of their patients received RBV whereas in our study only 81% received RBV. Our study showed a median decrease of 2.0 ng/mL in AFP (p<0.01), consistent with a recent study which also reported a decrease in post-treatment AFP levels when compared to pre-treatment levels [35]. In contrast to findings reported by Patton etal, we found a significant increase in BMI post-SVR24 compared to pre-treatment in patients treated with both IFN-containing and IFN-free regimens, p<0.01 [38]. During active hepatitis C infection, there is increased inflammation which results in hyper metabolism. We believe that the rise in BMI post-treatment is due to the decrease in inflammation.

**Fig 4:**
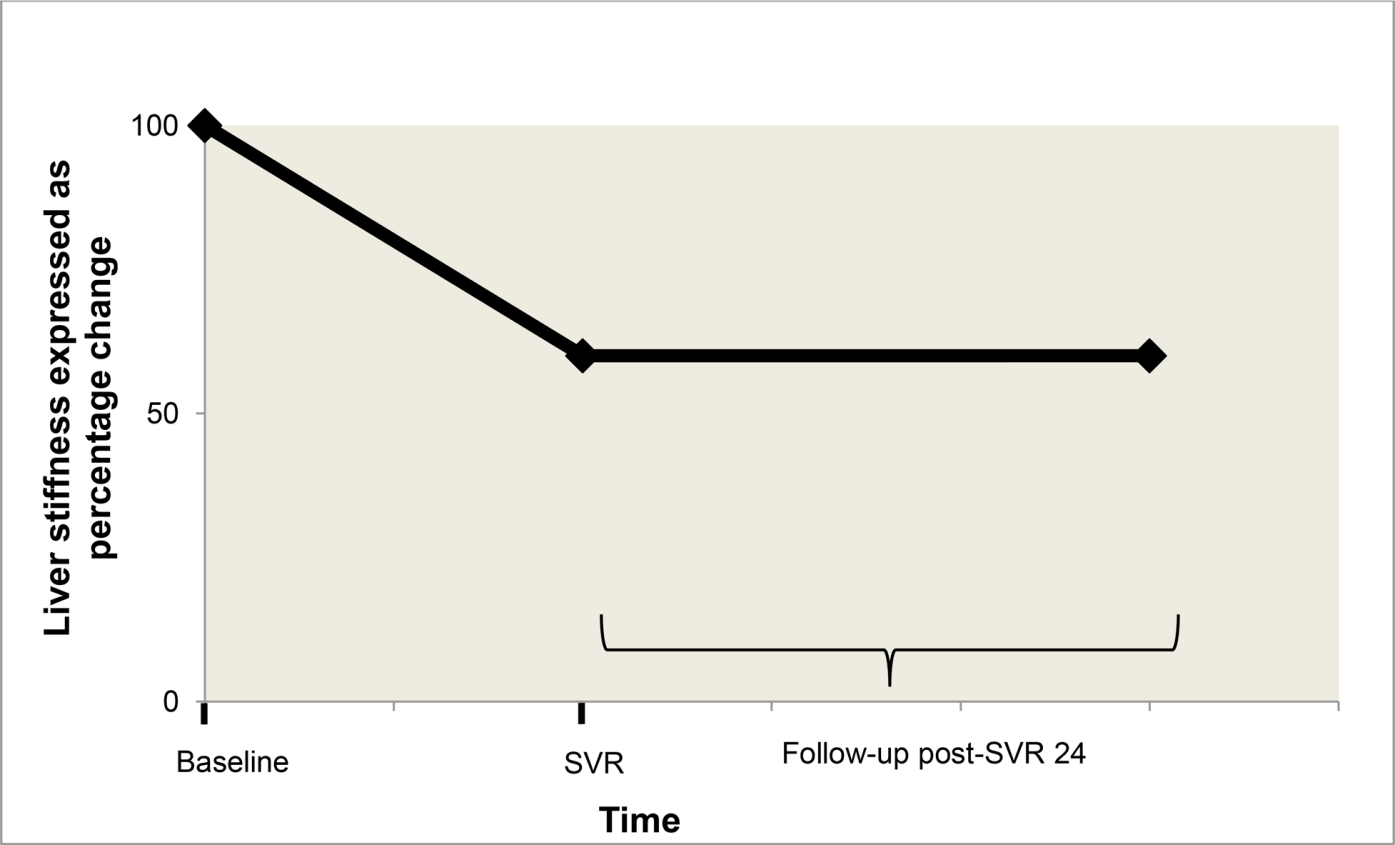
Model of changes in liver stiffness overtime. This model graph shows a percentage decrease in liver stiffness from pre-treatment to post-SVR24. The initial decline in liver stiffness is most likely due to regression of liver inflammation. After SVR24 there was little change in liver stiffness over time as indicated in Fig 3.

Our analysis of 56 patients with follow-up FibroScan data revealed a median LS score change (SVR24 to the end of follow-up) of -0.75 kPa, which was not statistically significant. Much of the decrease in LS (pre-treatment to SVR24) likely reflects resolution of liver inflammation [23]. Contrary to prior studies that show that fibrosis markers and liver histology improves significantly post-SVR [39, 40], we found that in the interval from SVR24 to the end of follow-up, there was minimal further reduction in liver-stiffness, which probably indicates that fibrosis regresses slowly post-SVR24 when it regresses at all (Fig 4). Treatment regimens for HCV are rapidly changing, and there is a great need for further studies on the effect of each regimen on liver fibrosis. Fibrosis regression has been shown to decrease the risk of HCC and mortality [14,41], but many patients are offered HCV treatment after fibrosis has been established. Since our study found persistently elevated LS in many patients, early diagnosis and treatment before fibrosis has been established is necessary to maximize the benefits of treatment. Twenty-eight of the 56 patients had cirrhosis prior to treatment and 16 had cirrhosis at the end of follow-up.

The strengths of this study include the information it provides on the impact of IFN-containing regimens and IFN-free regimens on LS and changes in LS during post-SVR24 follow-up. The limitations include the relatively small sample size of 100 patients, the lack of information about patients with HIV/HCV co-infection, and operator dependent variability with regard to performance of the FibroScan.

In conclusion, compared to pre-treatment values, SVR24 LS scores are significantly reduced; however, 66% percent of patients had SVR24 LS scores indicating ≥ F2 fibrosis (estimated using FibroScan) and cirrhosis persisted in approximately 60% of patients with cirrhosis pre-treatment. During a post-SVR24 follow-up period more than one year in duration, liver stiffness changed only minimally. Histological validation and increased follow-up time are needed to fully understand the impact of SVR on fibrosis stage and regression. There is an urgent need to identify and treat patients so that permanent liver damage can be avoided.

**Key Points**

- In many parts of the world, HCV treatment is prioritized for patients with significant liver damage (fibrosis) or co-morbid conditions.
- This study demonstrated that liver stiffness decreases between baseline and SVR24, but plateaus thereafter, suggesting that many patients who have advanced liver disease at the time they receive HCV treatment will have residual liver disease even if treatment leads to a sustained virological response (SVR).
- Patients who have advanced fibrosis or cirrhosis at the time they receive HCV treatment may be left with permanent liver damage.
- The lack of complete resolution of liver damage necessitates the early identification and treatment of HCV patients to prevent permanent liver damage.

## Supporting Information

S1 File Minimal Data Set of 100 Patients. (XLSX)
